# *Notes From the Field:* Overdose Deaths Involving Eutylone (Psychoactive Bath Salts) — United States, 2020

**DOI:** 10.15585/mmwr.mm7132a3

**Published:** 2022-08-12

**Authors:** R. Matt Gladden, Vaughne Chavez-Gray, Julie O’Donnell, Bruce A. Goldberger

**Affiliations:** ^1^Division of Overdose Prevention, National Center for Injury Prevention and Control, CDC; ^2^Oak Ridge Institute for Science and Education, Oak Ridge, Tennessee; ^3^Department of Pathology, Immunology and Laboratory Medicine, University of Florida College of Medicine, Gainesville, Florida.

Synthetic cathinones (known as psychoactive bath salts) are a class of potent central nervous stimulants that mimic the effects produced by cocaine, methamphetamine, and methylenedioxymethamphetamine (MDMA; known as ecstasy). Synthetic cathinones have been sold as MDMA (*1*), distributed as nondrug products (e.g., bath salts) to conceal their sale as an illicit drug and also sold as illicit drug products.* From 2017 to 2021, the supply of eutylone^†^ (a synthetic cathinone) rapidly increased in the United States. During January–June 2017, eutylone was detected in fewer than 10 drug items such as powders, capsules, or tablets obtained through law enforcement activities such as drug seizures, arrests, or undercover buys and tested; during January–June 2021, eutylone was detected in 8,379 drug items, making it the seventh most identified drug during this period (*2*). Public alerts have been issued and include concern about elevated overdose risk associated with eutylone being sold as MDMA^§^ (*1*). Little is known about the relative potencies and pharmacological profile of synthetic cathinones compared with MDMA, and using counterfeit tablets potentially increases the risk for overdose; however, additional investigation is needed.

CDC, through the State Unintentional Drug Overdose Reporting System (SUDORS), funds 47 states and the District of Columbia^¶^ to enhance postmortem toxicology testing and abstract comprehensive data from death certificates and medical examiner or coroner reports, including toxicology reports, for drug overdose deaths of unintentional and undetermined intent. This report describes overdose deaths in which the medical examiner or coroner determined that eutylone contributed to the death (eutylone-involved deaths), submitted to SUDORS by 43 states and the District of Columbia with data for January–June 2020, July–December 2020, or both.** For three states (Alabama, South Carolina, and Wisconsin), data from the death certificate only were analyzed. This activity was reviewed by CDC and was conducted consistent with applicable federal law and CDC policy.^††^

During 2020, 343 eutylone-involved deaths were reported by 22 of 44 SUDORS jurisdictions, with 259 (75.5%) concentrated in two southern states^§§^ (Florida [182] and Maryland [77]). Eutylone-involved deaths commonly co-involved illicitly manufactured fentanyls (IMFs)^¶¶^ (which include both illicitly manufactured fentanyl and fentanyl analogs) (77.3%), and cocaine or methamphetamine (53.1%) (Table). Among 183 (53.4%) of 343 eutylone-involved deaths with medical examiner or coroner reports available (from 41 of 44 jurisdictions),*** 23 (12.6%) had negative MDMA toxicology findings but evidence of MDMA use before the overdose or a history of MDMA use.^†††^ One of the 23 deaths was in a person who had a history of cathinone use.

**TABLE:** Demographic and other characteristics of drug overdose deaths involving eutylone (N = 343), by co-involvement with opioids — State Unintentional Drug Overdose Reporting System, United States,* 2020

Characteristic	No. (%) of eutylone-involved deaths		
	Total deaths	Deaths involving any opioid	Deaths not involving any opioid
**Total**	**343 (100.0)**	**283 (100.0)**	**60 (100.0)**
**Sex^†^**			
Male	246 (71.7)	203 (71.7)	43 (71.7)
Female	97 (28.3)	80 (28.3)	17 (28.3)
**Age group, yrs^†^**			
15–24	24 (7.0)	20 (7.1)	4 (6.7)
25–34	130 (37.9)	111 (39.2)	19 (31.7)
35–44	102 (29.7)	83 (29.3)	19 (31.7)
45–54	57 (16.6)	45 (15.9)	12 (20.0)
≥55	30 (8.7)	24 (8.5)	6 (10.0)
**Race and ethnicity^§^**			
White, non-Hispanic	161 (46.9)	144 (50.9)	17 (28.3)
Black, non-Hispanic	115 (33.5)	78 (27.6)	37 (61.7)
Other, non-Hispanic	8 (2.3)	8 (2.8)	0 (—)
Hispanic	37 (10.8)	34 (12.0)	3 (5.0)
Unknown/Missing	22 (6.4)	19 (6.7)	3 (5.0)
**U.S. Census Bureau region of the state^†,¶^**			
Northeast	14 (4.1)	10 (3.5)	4 (6.7)
Midwest	12 (3.5)	9 (3.2)	3 (5.0)
South	314 (91.5)	261 (92.2)	53 (88.3)
West	3 (0.9)	3 (1.1)	0 (—)
Drugs involved in overdose**			
Any opioid	283 (82.5)	283 (100.0)	—^††^
IMFs	265 (77.3)	265 (93.6)	—^††^
Heroin	39 (11.4)	39 (13.8)	—^††^
Prescription opioid	39 (11.4)	39 (13.8)	—^††^
Other stimulants, not eutylone^†^	191 (55.7)	164 (58.0)	27 (45.0)
Cocaine or methamphetamine^§^	182 (53.1)	159 (56.2)	23 (38.3)
Methamphetamine^†^	54 (15.7)	43 (15.2)	11 (18.3)
Cocaine^§^	147 (42.9)	133 (47.0)	14 (23.3)
No opioid or other stimulant	33 (9.6)	—^††^	33 (55.0)
Benzodiazepines^†^	48 (14.0)	44 (15.5)	4 (6.7)
**Total eutylone-involved deaths in 41 jurisdictions^§§^ with medical examiner/coroner data^¶¶^**	**183 (100.0)**	**151 (100.0)**	**32 (100.0)**
**Evidence of current or past MDMA use^§^**	23 (12.6)	10 (6.6)	13 (40.6)
**Evidence of MDMA use before overdose^§^**	13 (7.1)	4 (2.6)	9 (28.1)
**History of chronic MDMA use^†^**	15 (8.2)	8 (5.3)	7 (21.9)

**Abbreviations:** IMF = illicitly manufactured fentanyl; MDMA = methylenedioxymethamphetamine.

* Forty-four jurisdictions provided data: Alabama, Alaska, Arizona, Arkansas, Colorado, Connecticut, Delaware, District of Columbia, Florida, Georgia, Hawaii, Illinois, Iowa, Kansas, Kentucky, Louisiana, Maine, Maryland, Massachusetts, Michigan, Minnesota, Mississippi, Missouri, Montana, Nebraska, Nevada, New Hampshire, New Jersey, New Mexico, North Carolina, Ohio, Oklahoma, Oregon, Pennsylvania, Rhode Island, South Carolina, South Dakota, Tennessee, Utah, Vermont, Virginia, Washington, West Virginia, and Wisconsin. Data only from the death certificate were analyzed for three states: Alabama, South Carolina, and Wisconsin.

^†^ No significant difference between eutylone-involved deaths with and without opioids was found using Fisher’s exact test (p>0.05).

^§^ A significant difference between eutylone-involved deaths with and without opioids was found using Fisher’s exact test (p<0.05). Test excluded missing values.

^¶^ U.S. Census Bureau regions were used to stratify jurisdictions into geographic regions. https://www2.census.gov/geo/pdfs/maps-data/maps/reference/us_regdiv.pdf

** A drug overdose can involve multiple drugs, such as IMF, eutylone, and cocaine. Consequently, specific drug percentages when summed will exceed 100%.

**^††^** By definition, this category will be zero. For example, eutylone-involved deaths with no opioid co-involvement did not have any opioids (e.g., IMF, heroin, and prescription) involved in the overdose.

^§§^ Did not include Alabama, South Carolina, or Wisconsin. Only 26 of the 182 eutylone-involved deaths in Florida had a medical examiner report at the time of this analysis and thus are not representative of Florida eutylone-involved deaths.

^¶¶^ Two authors reviewed narrative information abstracted from medical examiner or coroner reports for evidence of decedent using MDMA before the overdose (i.e., witness reported MDMA use by decedent before overdose symptoms) or a history of MDMA use (i.e., decedent was known by family to use MDMA frequently).

In 2020, most eutylone-involved deaths occurred within two states in the South, the region with the most eutylone drug reports by law enforcement in both 2019 and 2020 (*2*). Rapid increases in drug products containing eutylone (*2*), coupled with the concentration of eutylone-involved deaths in a few states, warrant enhanced surveillance for new outbreaks in other states involving emerging or known synthetic cathinones, including eutylone. Starting in late 2021, the World Health Organization Expert Committee on Drug Dependence reviewed and then recommended legally regulating the international distribution of eutylone; subsequently, the United Nations Commission on Narcotics Drugs internationally scheduled eutylone with enforcement beginning on November 23, 2022.^§§§^ International scheduling of eutylone might be contributing to its replacement with a newer synthetic cathinone with sharp increases in N,N-dimethylpentylone and declines in eutylone reported in 2022.^¶¶¶^

Understanding whether eutylone exposure is intended or unintended (i.e., via adulterated substances) can guide prevention efforts. Consistent with previously reported unintentional exposure among persons using MDMA (*1*), approximately one in 10 eutylone-involved deaths in this report had evidence of current or past MDMA use but no toxicology finding of MDMA. Common co-involvement of IMFs in eutylone-involved deaths is consistent with the increased prevalence of concurrent use of IMFs with illicit stimulants (*3*). However, infrequent documentation of purposeful cathinone use in eutylone-involved deaths might indicate unintended exposures and needs further investigation. One half of eutylone-involved deaths co-involved cocaine or methamphetamine, which heightens fatal overdose risk because of the cumulative effects of multiple stimulants. This high level of co-involvement could be related to unintentional exposure or part of an increasing trend to co-use multiple stimulants such as methamphetamine and cocaine (*4*). Risk for unintentional eutylone exposure might be mitigated by 1) increasing knowledge about synthetic cathinones, including eutylone, among persons using MDMA and other drugs with eutylone, 2) supporting rapid dissemination of results from enhanced toxicology testing of illicit drug products, including those sold as MDMA, and 3) broadly increasing availability and access to harm reduction strategies.

## Footnotes

*
https://www.dea.gov/sites/default/files/2020-06/Bath%20Salts-2020.pdf

†
https://www.deadiversion.usdoj.gov/drug_chem_info/eutylone.pdf

§ https://www.npsdiscovery.org/wp-content/uploads/2020/03/Public-Alert_Eutylone_Benzylone_NPS-Discovery_033120.pdf; https://cdn.ymaws.com/www.fadaa.org/resource/resmgr/files/resource_center/trend_alert_4__eutylone_fada.pdf; https://cdn.who.int/media/docs/default-source/essential-medicines/unedited-advance-copy-44th-ecdd-critical-review-report-eutylone.pdf?sfvrsn=ca370181_3&download=true.

¶
https://www.cdc.gov/drugoverdose/fatal/sudors.html

** *January–December 2020:* Alaska, Arizona, Arkansas, Colorado, Connecticut, Delaware, District of Columbia, Florida, Georgia, Illinois, Kansas, Kentucky, Maine, Maryland, Massachusetts, Michigan, Minnesota, Mississippi, Missouri, Montana, Nebraska, Nevada, New Hampshire, New Jersey, New Mexico, North Carolina, Ohio, Oklahoma, Oregon, Pennsylvania, Rhode Island, South Dakota, Tennessee, Utah, Vermont, Virginia, Washington, and West Virginia; *January–June 2020:* Wisconsin; *July–December 2020:* Alabama, Hawaii, Iowa, Louisiana, and South Carolina.

†† 45 C.F.R. part 46; 21 C.F.R. part 56; 42 U.S.C. Sect. 241(d), 5 U.S.C. Sect. 552a, 44 U.S.C. Sect. 3501 et seq.

§§ U.S. Census Bureau regions were used to stratify jurisdictions into geographic regions (https://www2.census.gov/geo/pdfs/maps-data/maps/reference/us_regdiv.pdf). Analyses of overdose characteristics included the following 44 jurisdictions: eight of nine in the Northeast region; 10 of 12 in the Midwest region; 16 of 17 in the South region; and 10 of 13 in the West region.

¶¶ Fentanyl was classified as likely illicitly manufactured using toxicology, scene, and witness evidence. When evidence was insufficient to classify fentanyl as illicit or prescription, it was classified as illicit because most fentanyl overdose deaths involve illicit fentanyl. All fentanyl analogs except alfentanil, remifentanil, and sufentanil (which have legitimate human medical use) were included as IMFs.

*** Alabama, South Carolina, and Wisconsin were not included. Only 26 of 182 eutylone-involved deaths in Florida had a medical examiner report at the time of this analysis and thus are not representative of Florida eutylone-involved deaths.

††† Two authors reviewed narrative information abstracted from medical examiner or coroner reports for evidence of decedent using MDMA before the overdose (i.e., witness reported MDMA use by decedent before overdose symptoms) or a history of MDMA use (i.e., decedent was known by family to use MDMA frequently).

§§§ https://www.who.int/publications/i/item/9789240042834; https://www.unodc.org/LSS/Announcement/Details/a56e0bd9-0da5-4152-a34d-7cff7746bf50

¶¶¶
https://www.npsdiscovery.org/wp-content/uploads/2022/07/2022-Q2_NPS-Stimulants-and-Hallucinogens_Trend-Report.pdf
